# Suspected Pentalogy of Cantrell With Ectopia Cordis in a Newborn: Diagnostic and Management Challenges in a Low Resource Setting in Northern Tanzania

**DOI:** 10.1002/ccr3.73807

**Published:** 2026-10-28

**Authors:** Tabitha Mshanga, Lightness Chuwa, Kajal Jethwa, Ronald Mbwasi

**Affiliations:** ^1^ Department of Pediatrics and Child Health Kilimanjaro Christian Medical Centre (KCMC) Moshi Kilimanjaro Tanzania; ^2^ KCMC University Moshi Kilimanjaro Tanzania

**Keywords:** congenital anomaly, ectopia cordis, low‐resource setting, neonatal outcome, pentalogy of Cantrell, Tanzania

## Abstract

Suspected Pentalogy of Cantrell with ectopia cordis carries very high neonatal mortality. In low‐resource settings, absent antenatal ultrasound and limited access to imaging and pediatric cardiac surgery reduce diagnostic certainty and survival. Prenatal diagnosis does not guarantee survival, but enables parental counseling, multidisciplinary planning and delivery at an appropriately resourced center.

## 1 Introduction

Pentalogy of Cantrell (also known as Cantrell syndrome) is a rare congenital anomaly first described by Cantrell and colleagues in 1958 [1]. Globally, it is estimated to occur in approximately 5.5–7.9 per 1,000,000 live births [2]. Classically, the syndrome is defined by five major malformations involving (1) the midline supraumbilical abdominal wall, (2) the lower sternum, (3) the anterior diaphragm, (4) the diaphragmatic pericardium, and (5) intracardiac defects [1].

We report a newborn with a clinical and radiographic picture suggestive of Pentalogy of Cantrell, managed at a tertiary referral facility in the Northern Zone of Tanzania. Because echocardiography and computed tomography (CT) could not be performed owing to financial constraints, not all five classical components could be directly demonstrated; we detail which findings were directly observed, which were suspected, and which remained unassessed (Table 1). This case illustrates the profound clinical and diagnostic challenges posed by financial and infrastructural barriers in a low‐resource setting.

**TABLE 1:** Pentalogy of Cantrell—component verification.

S/No	Classical component	Status	Basis/Notes
1.	Sternal defect	Suspected	Sternum not appreciated on chest radiograph; possible sternal aplasia. Not confirmed by CT.
2.	Supraumbilical abdominal wall defect	Directly observed	8 × 10 cm membrane‐covered midline supraumbilical defect; umbilical cord attached to its left lateral margin; no specific herniated organs identified on clinical examination.
3.	Diaphragmatic pericardial defect	Unassessed	Echocardiography and CT were not performed.
4.	Anterior diaphragmatic defect	Unassessed	Echocardiography and CT were not performed.
5.	Intracardiac defect	Unassessed	No echocardiography performed.

## 2 Case Presentation

A two‐hour‐old female infant was referred to our neonatal intensive care unit (NICU) from a health center because of an externally visible, pulsating heart on the anterior chest wall and a supraumbilical midline abdominal defect. The infant was born at 38 weeks of gestation via spontaneous vaginal delivery to a 17‐year‐old primigravida. The baby cried immediately after birth, with Apgar scores of 9 and 10 at the first and fifth minutes respectively, and a birth weight of 2800 g. The mother had registered for antenatal care at 16 weeks of gestation and attended a total of four visits. She remained normotensive, normoglycemic, and HIV‐negative throughout pregnancy and received routine iron and folate supplementation and tetanus prophylaxis. No antenatal ultrasound imaging had been performed. The mother reported no known family history of congenital anomalies, no exposure to radiation or toxic chemicals, and did not live near an industrial area.

On admission the infant was alert, pink, and was in mild respiratory distress. Axillary temperature was 36.5°C, respiratory rate 55 breaths per minute, heart rate 138 beats per minute, and oxygen saturation 85% on 1 L/min of oxygen via nasal prongs.

## 3 Examination Findings

*Thorax*: A sternal defect with the heart exposed and actively pulsating on the midline chest wall, lacking pericardial or skin cover (ectopia cordis).

*Abdomen*: A midline supraumbilical abdominal wall defect measuring approximately 8 × 10 cm, covered by a thin membrane, with the umbilical cord attached to its left lateral margin. No specific viscera were identified as herniated on clinical examination.

*Other systems*: Primitive reflexes (sucking, grasping, palmar, plantar) were intact, with normal external female genitalia and a patent anus.

Because echocardiography and CT were not performed, a definitive Toyama classification could not be established. A provisional clinical diagnosis of suspected Pentalogy of Cantrell with ectopia cordis was made, based on directly observed ectopia cordis, suspected sternal defect, and a directly observed supraumbilical abdominal wall defect. (Figure 1).

**FIGURE 1:**
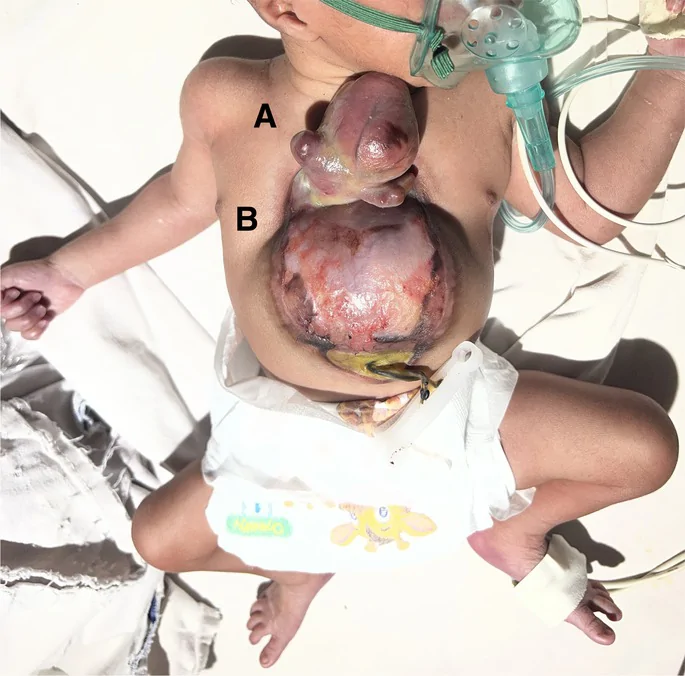
Clinical photograph showing (A) the ectopic heart exposed through the sternal defect and (B) an adjacent membrane‐covered supraumbilical abdominal wall defect, measuring 8 cm by 10 cm with the umbilical cord visible at its left lateral margin. No specific herniated viscera were identified on clinical examination.

### 3.1 Management and Clinical Course

The neonate was nursed in the NICU under a radiant warmer with minimal handling to maintain normothermia. A pediatric cardiologist was locally consulted and reviewed the neonate. Oxygen therapy was initiated via face mask. The exposed thoracic and abdominal wall defects were covered with sterile, moist normal‐saline gauze changed twice daily to preserve tissue hydration and minimize contamination risk. A neonatologist and pediatric cardiologist from the potential referring center were consulted. Empirical intravenous antibiotic therapy (ampicillin/cloxacillin and gentamicin) was started, alongside fluid management (10% dextrose in water) and analgesia (paracetamol). Total parenteral nutrition was recommended for this patient basing on the clinical condition, however due to unavailability at our setting hence we opted for trophic feeds via nasogastric tube, which was initiated to sustain the nutritional needs of the neonate.

A plain chest radiograph on day of life 2 showed non‐visualization of the left lung field, a clear right lung field and unappreciated sternum; these findings were read as consistent with a suspected left lung hypoplasia (Figure 2). Thoracic CT and echocardiography were urgently recommended the same day to evaluate the intracardiac and diaphragmatic anatomy and to confirm the extent of pulmonary hypoplasia, but due to both the caregivers' severe financial constraints and the opinion of the pediatric cardiologist that definitive surgery was unlikely for the neonate, cost exemptions were not sought. Similarly, urgent referral to the Jakaya Kikwete Cardiac Institute (JKCI) in Dar es Salaam was initially proposed for specialized pediatric cardiothoracic surgical evaluation but remained unfeasible, as there was no guarantee of definitive management, and with the higher cost of inter‐facility transportation and the anticipated cost of care at the receiving institution, it was mutually decided with the parents not to transfer the neonate and to manage conservatively.

**FIGURE 2:**
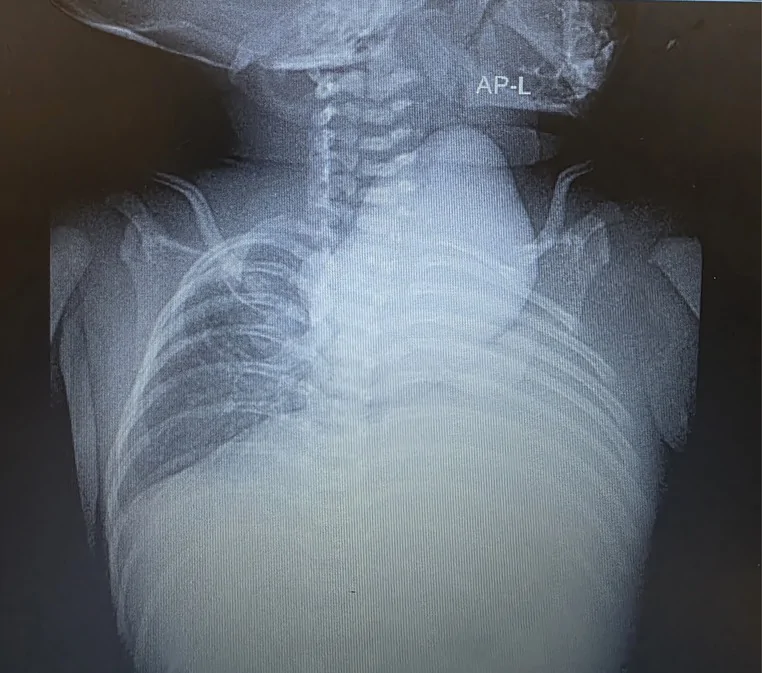
Anteroposterior chest radiograph showing the bony thorax with a large central mediastinal/cardiac silhouette and non‐visualization of the left lung field, with normal costophrenic and cardio phrenic angles and a clear right lung field; findings are consistent with suspected left lung hypoplasia.

Over the next 72 h the infant's condition progressively deteriorated. She developed worsening respiratory distress characterized by tachypnoea (up to 69 breaths/min), intercostal retractions, grunting, and nasal flaring, with oxygen saturation persistently dropping into the 65%–80% range despite supplemental oxygen. On the fourth day of life, the neonate suffered acute cardiorespiratory collapse. Death was certified, with the immediate cause recorded as cardiorespiratory collapse in the setting of suspected pentalogy of Cantrell with severe ectopia cordis, and suspected pulmonary hypoplasia (Table 2).

**TABLE 2:** Clinical timeline.

Prenatal	No documented antenatal ultrasound at any point during the pregnancy.
At Birth	Spontaneous vaginal delivery at 38 weeks gestational age at a Health center with the Apgar score of9/10. Ectopia cordis and abdominal wall defect immediately obvious; Neonate was referred to our facility.
DOL 1	Neonate received at our facility. Ectopia cordis and abdominal wall defect immediately obvious; neonate transferred to NICU. Exposed heart and abdominal defect covered with sterile, moist saline gauze; neonate nursed under a radiant warmer in the supine position. Neonatologist and pediatric cardiologist were involved locally and at the referring hospital. Enteral feeding was initiated as trophic feeds via Nasogastric tube because total parenteral nutrition was unavailable at our center at the time, hence close monitoring was done and slow increases of trophic feeds via nasogastric tube were done to provide minimal nutritional needs.
DOL 2	Chest radiograph performed: externally located heart with superiorly positioned apex; left lung field not visualized; right lung field clear; sternum not appreciated (possible sternal aplasia). HRCT and echocardiography recommended but not performed. Referral to Jakaya Kikwete Cardiac Institute (JKCI) recommended; referral was limited by not guarantee of survival after management, transportation costs and anticipated costs of care at the receiving facility. Hence it was mutually decided with the parents not to refer the neonate and to manage conservatively.
DOL 3	Progressive respiratory deterioration (tachypnoea, intercostal retractions, grunting, nasal flaring; oxygen saturations persistently 65%–80% despite supplemental oxygen).
DOL 4	Acute cardiorespiratory collapse and death.

## 4 Discussion

Pentalogy of Cantrell is an embryonic developmental defect believed to result from an abnormality in the differentiation and development of the ventral mesoderm between 14 and 18 days of gestation [1]. Disruption of mesodermal segment development leads to diaphragmatic, pericardial, and intracardiac lesions, whereas failure of the paired primordial structures to migrate centrally results in sternal and abdominal wall malformations [3]. In 1972, Toyama proposed a clinical classification based on the expression of defects [4]:

Class 1 (Definite Diagnosis): Presence of all five major defects.

Class 2 (Probable Diagnosis): Presence of four defects, including intracardiac and abdominal wall lesions.

Class 3 (Incomplete Expression): Presence of various combinations of defects, always including a sternal abnormality.

Our patient's presentation is most consistent with an incomplete expression ectopia cordis and a supraumbilical abdominal wall defect were directly observed, a sternal defect was suspected on examination and radiography, and pulmonary hypoplasia was suspected on plain radiography. Because echocardiography and CT were not performed, the diaphragmatic, pericardial, and intracardiac components could not be assessed, and the classification should be regarded as provisional rather than definitively established (Table 1).

In ideal settings, early prenatal diagnosis via routine ultrasound or fetal magnetic resonance imaging is important [5]. Antenatal screening allows parents to receive multidisciplinary counseling and facilitates delivery planning at tertiary pediatric surgical centers equipped with advanced neonatal cardiopulmonary support [3]; however, it does not guarantee survival in severe ectopia cordis. As highlighted in this case in which no antenatal ultrasound was performed at any point in the pregnancy and echoed in similarly resource‐constrained presentations elsewhere [6], prenatal diagnosis is frequently missed in low‐resource settings where routine antenatal ultrasonography is either unavailable or unaffordable, independently limiting any opportunity for anticipatory delivery planning.

Beyond prenatal diagnosis, postnatal diagnostic evaluation (CT, MRI, and echocardiography) and definitive surgical repair are severely restricted by financial and capacity‐related barriers in this setting, directly influencing neonatal outcomes. Where these barriers are absent and surgical correction is feasible, outcomes can be markedly better, as demonstrated by a recent report of successful chest‐wall reconstruction and long‐term normal development following ectopia cordis repair [7]. The more distinctive contribution of this case is therefore not the anomaly itself, which is well described, but the practical diagnostic, multidisciplinary, and referral challenges it illustrates in a resource‐limited setting.

## 5 Conclusion

Suspected Pentalogy of Cantrell is an exceptionally rare and complex congenital presentation associated with high neonatal mortality, particularly with ectopia cordis and pulmonary hypoplasia. This case underscores the value of accessible prenatal ultrasound screening for early detection and multidisciplinary counseling and planning, while recognizing that prenatal diagnosis alone does not guarantee survival. It also highlights the health‐system and financial barriers facing families in resource‐limited settings, where limited access to subsidized pediatric cardiothoracic care and advanced diagnostic imaging constrains both diagnostic certainty and definitive management in neonates with complex surgical conditions.

## Author Contributions

**Tabitha Mshanga:** conceptualization, writing – original draft, writing – review and editing. **Lightness Chuwa:** conceptualization, writing – review and editing, methodology. **Kajal Jethwa:** conceptualization, writing – review and editing, methodology. **Ronald Mbwasi:** conceptualization, supervision, writing – review and editing.

## Funding

The authors have nothing to report.

## Ethics Statement

This case report was reviewed and approved for publication at the departmental level by the Department of Pediatrics and Child Health, Kilimanjaro Christian Medical Centre (KCMC), Moshi, Tanzania. This approval did not constitute review by a formal Institutional Review Board or Research Ethics Committee.

## Consent

Written informed consent was obtained from the child's mother for publication of this case report and any accompanying clinical images. A copy of the written consent is available for review by the Editor‐in‐Chief of this journal on request.

## Conflicts of Interest

The authors declare no conflicts of interest.

## Data Availability

Data sharing is not applicable to this article as no datasets were generated or analyzed. All data supporting the findings are included within the article.
